# Information Seeking in Social Media: A Review of YouTube for Sedentary Behavior Content

**DOI:** 10.2196/ijmr.3835

**Published:** 2015-01-20

**Authors:** Emily Knight, Brittany Intzandt, Alicia MacDougall, Travis J Saunders

**Affiliations:** ^1^University of Western OntarioHealth and Rehabilitation SciencesLondon, ONCanada; ^2^Wilfrid Laurier UniversityWaterloo, ONCanada; ^3^University of Western OntarioKinesiologyLondon, ONCanada; ^4^University of Prince Edward IslandFaculty of ScienceCharlottetown, PECanada

**Keywords:** sedentary lifestyle, translational medical research, social media

## Abstract

**Background:**

The global prevalence of sedentary lifestyles is of grave concern for public health around the world. Moreover, the health risk of sedentary behaviors is of growing interest for researchers, clinicians, and the general public as evidence demonstrates that prolonged amounts of sedentary time increases risk for lifestyle-related diseases. There is a growing trend in the literature that reports how social media can facilitate knowledge sharing and collaboration. Social sites like YouTube facilitate the sharing of media content between users.

**Objective:**

The purpose of this project was to identify sedentary behavior content on YouTube and describe features of this content that may impact the effectiveness of YouTube for knowledge translation.

**Methods:**

YouTube was searched on a single day by 3 independent reviewers for evidence-based sedentary behavior content. Subjective data (eg, video purpose, source, and activity type portrayed) and objective data (eg, number of views, comments, shares, and length of the video) were collected from video.

**Results:**

In total, 106 videos met inclusion criteria. Videos were uploaded from 13 countries around the globe (ie, Australia, Barbados, Belgium, Canada, Colombia, Kenya, New Zealand, Russia, South Africa, Spain, Ukraine, United Kingdom, United States). The median video length was 3:00 minutes: interquartile range (IQR) 1:44-5:40. On average, videos had been on YouTube for 15.0 months (IQR 6.0-27.5) and had been viewed 239.0 times (IQR 44.5-917.5). Videos had remarkably low numbers of shares (median 0) and comments (median 1). Only 37.7% (40/106) of videos portrayed content on sedentary behaviors, while the remaining 66 videos portrayed physical activity or a mix of behaviors. Academic/health organizations (39.6%, 42/106) and individuals (38.7%, 41/106) were the most prevalent source of videos, and most videos (67.0%, 71/106) aimed to educate viewers about the topic.

**Conclusions:**

This study explored sedentary behavior content available on YouTube. Findings demonstrate that there is confusion between physical activity and sedentary behaviors, that content is being uploaded to the site from around the globe, that content is primarily from health organizations and individuals with the purpose of educating fellow users, but that low views, comments, and shares suggest that sedentary behavior content is relatively underutilized on YouTube. Future research may wish to leverage social platforms, such as YouTube, to facilitate implementation and sharing of evidence-based sedentary behavior content.

##  Introduction

### Sedentary Behavior

Since the 1950s, researchers have understood the importance of physical activity in promoting health [1]. An active lifestyle is now understood to reduce both the morbidity and mortality of a wide range of chronic diseases ranging from cancer to cardiovascular disease [2]. Insufficient levels of physical activity are responsible for 6% of global mortality and are the fourth leading cause of death around the world [3].

While the health importance of physical activity is well established, recent evidence suggests that sedentary behavior (eg, activities done while sitting) [4] also plays an important role in the development of chronic disease. For example, sedentary behaviors are associated with adverse health effects, including undesirable changes in cardiometabolic markers, vascular, bone, and psychosocial health independent of exercise [5-8]. This is important, given that it is possible to accumulate high amounts of both sedentary and exercise behaviors during a single day [6,7,9-11]. Further, the proportion of time spent in sedentary behavior dwarfs that spent in physical activity. For example, Canadian adults spend roughly 70% of their waking hours engaging in sedentary behavior, while just 3% engaged in moderate or vigorous physical activity [12]. Not surprisingly, recent studies have estimated that sedentary behavior may reduce the life expectancy of Western nations by 1-2 years [13,14]. Given these important differences, researchers have recently argued that sedentary behavior should be viewed as an independent and distinct construct, rather than simply the lack of physical activity [4]. Further, it has been shown that sedentary behavior and physical activity have independent and distinct relationships with health.

### Knowledge Translation

The knowledge-to-action framework from Graham et al [15] highlights the multifaceted nature of knowledge creation and implementation. Knowledge about the health effects of prolonged sedentary behaviors has been generated (ie, the knowledge creation cycle). However, a gap exists in understanding how this information is being implemented to impact the health of lay users (ie, the knowledge utilization cycle).

In North America, the Internet is a primary source of health information, with more than half of users seeking health information online [16-18]. Previous evidence has reported that over half of American and European citizens have used the Internet to seek health-related information [19]. Online mediums may help to bridge the research to action gap by allowing evidence-based information to freely reach the homes of a broad spectrum of users. There is a growing trend in the literature reporting how social media can facilitate knowledge sharing and collaboration [20].

### YouTube

YouTube is among the top three most popular websites visited around the world, with more than 4 billion videos being watched by users daily [21,22]. The primary concept of social media sites like YouTube is the sharing of media content between users [23]. Videos allow for the sharing of complex ideas in a simple format [22]. The Health Care Social Media List form the Mayo Clinic identifies over 700 health-related associations in the United States that have established a presence on YouTube [24]. A strength of the social media format for knowledge translation is its capacity for timely updates, in contrast to the slow uptake and evolution of information shared through traditional peer-reviewed formats [19]. However, there is a lack of regulation on the content available through YouTube. Previous research has reported that misleading information posted through videos on YouTube could endanger viewers [22].

### Purpose

There is a growing body of evidence exploring health content available through YouTube. Research has identified that health-related videos posted to YouTube may contain erroneous and potentially harmful health information [20,22,25]. As knowledge about the health risk of sedentary behavior transitions from research to practice, there is value in understanding if the information available on YouTube is evidence-based. Therefore, the purpose of this project was to identify sedentary behavior content on YouTube and describe features of this content that may impact the effectiveness of YouTube for knowledge translation, such as evidence-informed messages and description of video characteristics. Understanding what information is currently available may help researchers tailor their messages to promote more effective knowledge translation and uptake.

## Methods

### Search Strategy

YouTube was searched on a single day (May 25, 2014) by 3 independent reviewers (EK, BI, AM). Computers were set to “incognito”/“worldwide” to limit any filtering by the site to previous user data and to help ensure that search results would not be limited to local country of searching but instead include videos from around the globe.

Similar to the methods of Williams et al [26], Google AdWords [27] was used to search keyword phrases frequently used by the public when searching Google in relation to “sedentary behavior” to develop a list of popular phrases for the topic that could be searched on YouTube. Subsequently, three keyword searches of YouTube were conducted (“sedentary behaviour”, “sedentary behavior”, and “sedentary lifestyle”) as well as a category search of the YouTube-generated “sedentary lifestyle” topic channel. Currently, YouTube algorithms create topic channels based on volume of content on the site, in the present case linked to the keyword phrase “sedentary lifestyle”. The channel’s main page presents three popular videos sorted by relevance to the topic channel for six subcategories: (1) popular sedentary lifestyle videos, (2) popular sedentary lifestyle and health videos, (3) popular sedentary lifestyle and physical exercise videos, (4) popular sedentary lifestyle and obesity videos, (5) popular sedentary lifestyle & lifestyle videos, and (6) popular sedentary lifestyle and childhood obesity videos.

For each of the four searches (three keyword, one topic channel), results were sorted by both relevance and views using the YouTube search features, and the first two result pages (approximately 40 videos) were assessed. The goal of the search process was to identify YouTube content that users are accessing the most. Searching was limited to the initial two pages of results based on the following a priori criteria: (1) saturation of the topic becomes evident in most cases by the end of the second page at which point search results are obviously not related to the search phrase, and unlike the searching process employed in systematic reviews, (2) typical YouTube users may be less likely to continue scanning results past the initial two pages of results, especially when videos appear not to be linked to the search phrase. Videos were excluded if they were not available in English or portrayed content obviously not related to the search (eg, music videos, product advertisements). Additionally, individual users’ channels that were generated in the search results were not reviewed, as they contained multiple videos posted by the user with varying relation to the desired search content.

### Data Management and Analysis

Video data were coded into an electronic spreadsheet and analyzed in June 2014. For each video, we collected both objective data (video title, URL, number of views, number of shares, the length of the video, number of comments posted by YouTube users, descriptive text and keywords that the user who uploaded the video included, and the YouTube category used to classify the video) and subjective data (the purpose and source of the video, and the type of activity content included in the video). Textbox 1 defines coding themes used for subjective data. To ensure consistency in coding, a representative video from each search was coded collaboratively until consensus was reached.

Descriptive statistics were performed to understand the context of evidence available on YouTube. Specifically, there was interest in understanding if the information available on YouTube represents best evidence for sedentary behavior, if users are seeking information on sedentary behavior through this medium, and describing video content in terms of who is producing the video to help inform future initiatives for leveraging YouTube as a knowledge translation vehicle. To explore the descriptive statistics by popularity of content on the YouTube site, interquartile range of view counts were used to group videos based on number of views. Pearson correlation coefficient (*r*) was calculated to explore the relationship between views, keyword search, video length, video source, and activity classification. Statistical significance was set at *P*<.05.

**Definition of coding themes for subjective data extraction.**

Video Source

- Media: Video presented by an identified news/media source
- Individual: Video presented by an individual
- Academic/Health Organization: Video presented by an academic conference, research group, or medical organization
- Consumer: Video endorsing and/or promoting sale of a product/service

Video Purpose

- Educate: Video informs/teaches about the topic, which includes evidence-based information
- Opinion: Video portraying an individual’s or organization’s perspective on the topic
- Academic Presentation: Video of a presentation to academic audiences (eg, conference proceedings)
- Commercial: Video promoting a company’s or individual’s product(s)

Activity Classification

- Physical Activity: Video portraying information on physical activity and the health benefits and/or public health recommendations for this activity
- Sedentary Behavior: Video portraying information on the behavior and/or health outcomes of activities in a sitting or reclining posture
- Mixed: Video incorporating information of both physical activity as well as activities in sitting/reclining postures

##  Results

**Figure 1:**
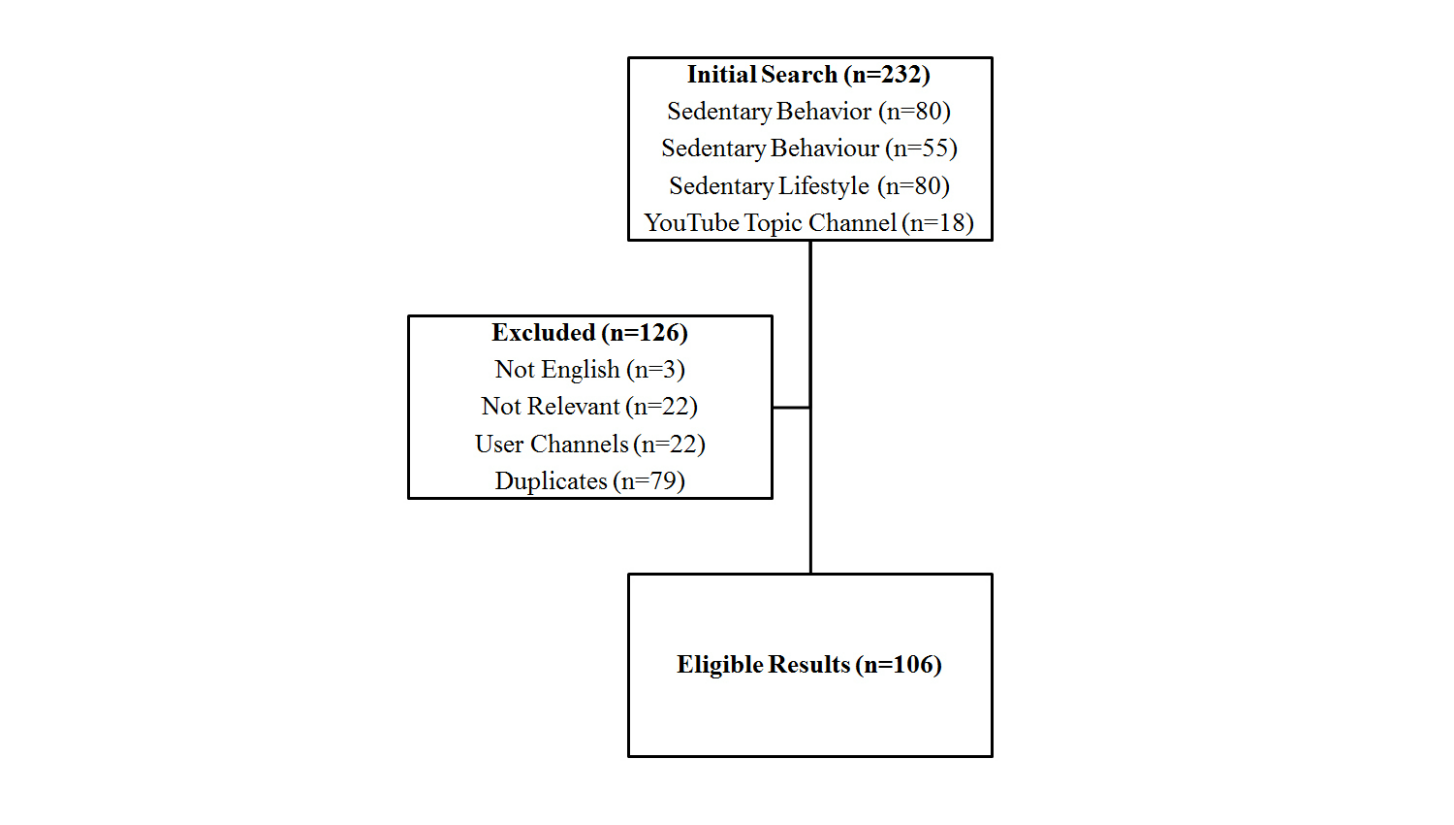
Search results.

**Figure 2:**
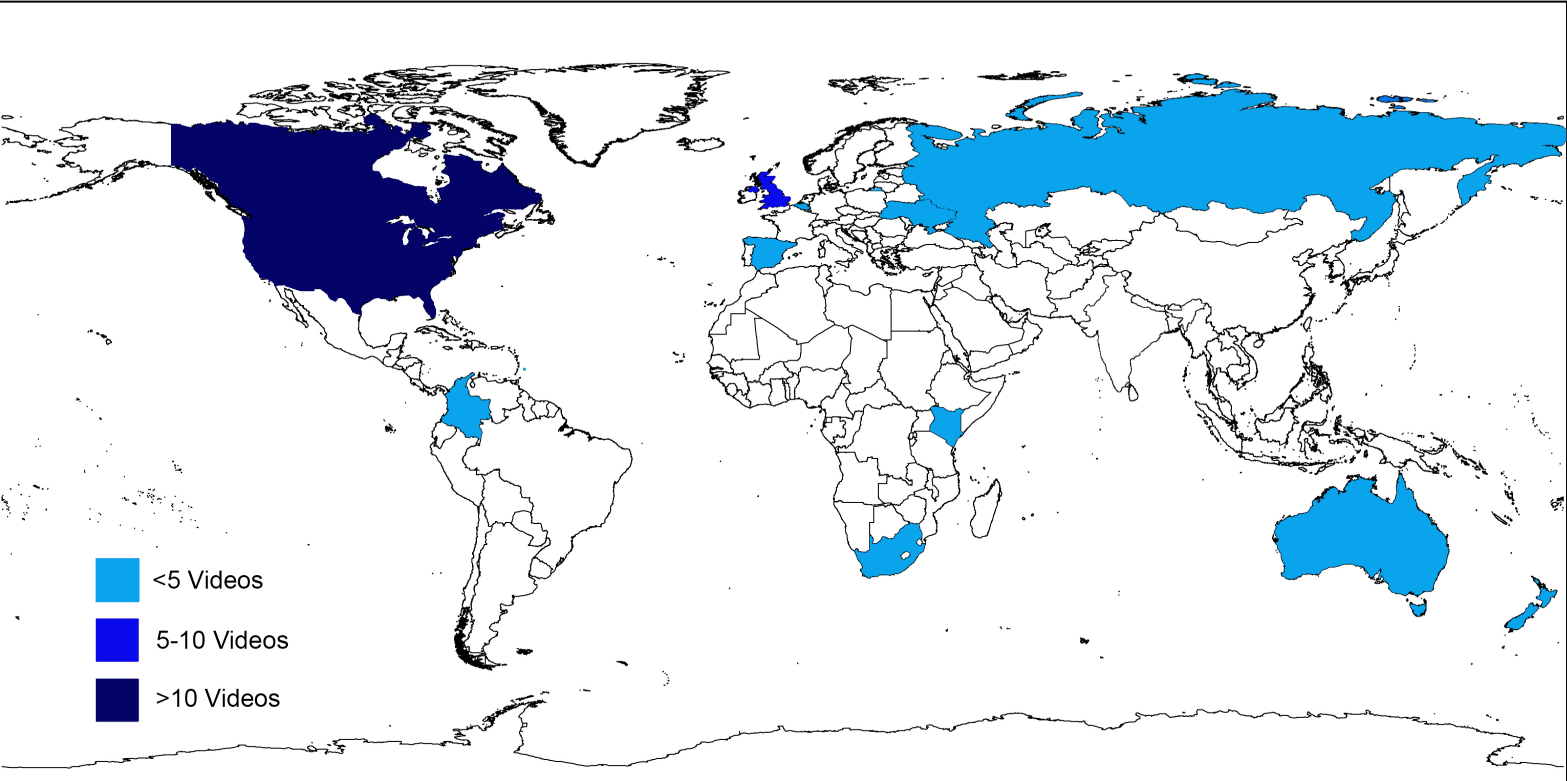
Global distribution of search results.

Figure 1 shows the search results. The initial search yielded 232 videos. After removing ineligible and duplicate results, data were analyzed from 106 videos. Table 1 summarizes the included videos. The location of the uploader was not discernable via the YouTube site. Therefore, an independent online database (YouTube Stats [28]) was used to search the country of origin for videos. It should be noted that the database provides only a source of origin for videos with >4 subscribers; therefore, it did not provide a complete source for origin of all videos in this sample (Table 2). Figure 2 shows the distribution of videos around the globe and demonstrates a substantial representation of content from North America.

**Table 1:** Video results.

Result No.	URL	Title	Months on YouTube	Length (min)	Views
1	http://www.youtube.com/watch?v=dJY9NWoA3Dk	10 Minute Basic Workout for the Sedentary Individual	19	3:40	495
2	https://www.youtube.com/watch?v=o12kXL0iopE	10-1. Sedentary Lifestyle and How To Improve Cardiovascular Endurance with Exercise	61	6:39	13,100
3	http://www.youtube.com/watch?v=oWW-Ws32MLI	15. Osteoporosis Sedentary Lifestyle	10	1:30	19
4	http://www.youtube.com/watch?v=YaucXroi8ls	2012 JustStand Wellness Summit; Dr David Dunstan, Baker IDI Heart and Diabetes Institute	9	59:41	67
5	http://www.youtube.com/watch?v=lJG4T5LpDzM	2014 Physical Activity Forum - Get Up, Stand Up	0	1:03:03	12
6	https://www.youtube.com/watch?v=6uomlJh5g9g	8 Weight Loss Tips for a Sedentary Lifestyle	23	3:16	65
7	http://www.youtube.com/watch?v=aoMmpFbZz-k	American Idle: Sedentary Time and Health	1	3:28	0
8	https://www.youtube.com/watch?v=MWVv0Z3x_xA	America's Walking | A Call to Action | A Sedentary Lifestyle	50	8:54	913
9	https://www.youtube.com/watch?v=3WxP7fU_JF4	An Introduction to Active vs. Sedentary Lifestyles as it Relates to Chronic Disease.wmv	50	2:05	3822
10	http://www.youtube.com/watch?v=sAo_352QYjs	Are you Active or Sedentary? How can you become more Active for FREE? Easy tips Lisa in Marbella	2	4:30	54
11	https://www.youtube.com/watch?v=NndqoguNrzs	Avoid Sedentary Lifestyle	2	6:40	14
12	http://www.youtube.com/watch?v=hMegW5G7ZEk	Back Pain & Sedentary Life Style	19	4:58	258
13	http://www.youtube.com/watch?v=tnQ1Ye6J5Aw	Beating Sedentary Behavior at Prince of Wales school	11	1:39	150
14	http://www.youtube.com/watch?v=clZq8w2lRHs	Benefits of a Standing Desk on your Feet Australia Campaign	3	5:25	90
15	http://www.youtube.com/watch?v=apEYRbfVsks	Bonnie Spring: Can we Design our Way out of the Obesity Epidemic?	31	42:39	132
16	http://www.youtube.com/watch?v=63SMM8tTELw	Bouncing at Work	9	0:47	101
17	https://www.youtube.com/watch?v=B9jhqaXZR0w	Breaking the Sedentary Lifestyle	10	2:52	17
18	http://www.youtube.com/watch?v=uqviPmuytQA	Breaks in Sedentary Time are Associated with Reduced Health Risk in Children.	5	4:18	128
19	https://www.youtube.com/watch?v=5ty7GhKJ0Yg	Camp Abilities: A Vision of a Healthier Lifestyle	46	3:08	1079
20	http://www.youtube.com/watch?v=UFg4amY6ltg	Classroom & Sedentary Behavior	6	6:49	53
21	http://www.youtube.com/watch?v=CLSbS0yEJ5M	Classroom Teacher Challenges for Managing PA: Reduce Sedentary Behavior with Strctu	21	1:21	922
22	https://www.youtube.com/watch?v=d4g7bPS_8pk	Combatting a Sedentary Lifestyle - Penn State Hershey	12	1:00	517
23	http://www.youtube.com/watch?v=osnXMZg4ccQ	Course Director Pitch - BSc Hons Physical Activity Exercise and Health	0	2:23	44
24	http://www.youtube.com/watch?v=sSKF0RoAOUY	Dan Oliver and Ryan Durden's Video Presentation	1	4:46	12
25	http://www.youtube.com/watch?v=lyBKXKBei8o	Dangers of a Sedentary Lifestyle	12	8:06	461
26	https://www.youtube.com/watch?v=to8GmlDfhmw	Dangers of a Sedentary Lifestyle	26	1:20	718
27	http://www.youtube.com/watch?v=eHoVLMFboBI	DynaCubes Breaking Sedentary behavior	11	1:36	32
28	https://www.youtube.com/watch?v=YRJpfkqYBp4	Easy Ways to Increase Physical Activity	49	0:58	12,298
29	https://www.youtube.com/watch?v=rasZGZpQsy0	Educating the Student Body: Taking Physical Activity and Physical Education to School	12	2:40	10,810
30	http://www.youtube.com/watch?v=B7r6r9UOxu0	Effect of Physical Activity on Serum Prostate-Specific Antigen Concentrations	16	5:05	117
31	http://www.youtube.com/watch?v=WwOdG1INtV8	EPI-NPAM 2012- Sedentary Behavior, Physical Activity and Incident Coronary Heart Disease	26	7:26	366
32	https://www.youtube.com/watch?v=PZVknpDsoNY	Erin has been Overweight her Whole Life	52	3:42	2003
33	http://www.youtube.com/watch?v=Iz0JgVoEFHc	Exercise & Weight Loss - Episode 7 - Summer Tomato Live	36	43:12:00	1343
34	http://www.youtube.com/watch?v=QFc-5oXgbYY	Exercise training Alters Subcutaneous White Adipose Tissue (scWAT)	11	2:18	684
35	http://www.youtube.com/watch?v=1PvQjNF2ths	Fitness Paradigm	21	43:25	56
36	https://www.youtube.com/watch?v=IqWfxxuxmi4	Give Up Your Sedentary Lifestyle	37	2:37	594
37	http://www.youtube.com/watch?v=Z_cx-n_7mXg	Gregory Norman - Physical Activity and Sedentary Behavior Classification Using Motion Sensor and SM	7	24:15	38
38	https://www.youtube.com/watch?v=Bxt0fplopvA	Health & Fitness Tips For Truck Drivers Revealed By Twin Drivers	4	1:08	1434
39	https://www.youtube.com/watch?v=P5ve869jb_Y	Healthy Eating vs. Sedentary Lifestyle - Fabio Viviani	12	4:55	1201
40	http://www.youtube.com/watch?v=A7vQWf-miVs	HK200 Ken Etics	2	2:06	1991
41	https://www.youtube.com/watch?v=5Nj3smpfUtM	How to Avoid a Sedentary Lifestyle:	46	1:47	5964
42	https://www.youtube.com/watch?v=PkpjnGHeNN4	How to Pronounce Sedentary	14	0:20	2151
43	https://www.youtube.com/watch?v=aDRYEYSb_f8	Informative Speech-Sedentary Lifestyle-Wilkey	13	7:42	30
44	http://www.youtube.com/watch?v=6BYvKdiWtcw	Intro.wmv	0	2:41	13
45	https://www.youtube.com/watch?v=ovAev4W7BeY	Is Korea Affected by an Abdominal Obesity and Sedentary Lifestyle Epidemic?	15	0:37	45
46	http://www.youtube.com/watch?v=4MnQ7XnBpcc	Jigsaw Desks	49	2:23	119
47	https://www.youtube.com/watch?v=2Zquq7I_Ol0	Joe Rogan on Fresh Food and a Sedentary Lifestyle	2	5:42	328
48	http://www.youtube.com/watch?v=BcxBeVaGnjI	Keynote Speech - Dr Kong Chen - Be Active 2012	18	50:16	147
49	http://www.youtube.com/watch?v=Mbr7rDe7vRw	L3 Health Online task Intro.wmv	30	2:20	27
50	https://www.youtube.com/watch?v=TOT6T--70_w	Lesson 2, The Benefits of a Healthy Lifestyle	30	2:10	741
51	https://www.youtube.com/watch?v=oh40z8MOzh0	Lifestyle: Ageing and Health	25	3:43	1866
52	http://www.youtube.com/watch?v=pH_iFV3nYnQ	Motivations for Continued Involvement in Physical Activity	22	4:33	348
53	http://www.youtube.com/watch?v=ElDA0YzORjs	Obesity: A Heavy Burden	4	1:07:52	239
54	http://www.youtube.com/watch?v=Ok96iSLWyyg	Older Women Spend 2/3rd of Time Sedentary	3	0:33	10
55	http://www.youtube.com/watch?v=3fV91ZGAkR0	Physical Activity and Sedentary Jobs	36	4:47	406
56	http://www.youtube.com/watch?v=XTTCKd8pZNQ	Prof. Stuart Biddle - Teaser	7	0:50	23
57	http://www.youtube.com/watch?v=yLJo5VECe5E	Project Play: Reimagining Youth Sports in America	3	1:14:19	4459
58	http://www.youtube.com/watch?v=9wRDIXxBmIY	Promoting More Physical Activity and Less Sedentary Behaviour in Young People	7	0:27	214
59	https://www.youtube.com/watch?v=L2E8_MJsNZA	PSA Sedentary Lifestyle	24	5:35	513
60	http://www.youtube.com/watch?v=hmCIuFv05ag	Scottish Kids Less Active	0	2:34	2
61	http://www.youtube.com/watch?v=ZuAKXAGZK0w	Sedentary Behavior	25	7:22	37
62	http://www.youtube.com/watch?v=qondXFSjPPA	Sedentary Behavior and your Health	5	0:51	31
63	http://www.youtube.com/watch?v=znXimY_iNvs	Sedentary Behavior in College Students	10	0:55	28
64	http://www.youtube.com/watch?v=Dr226ZCZuPw	Sedentary Behavior in Youth	6	5:11	24
65	http://www.youtube.com/watch?v=ynfUyMN6ReQ	Sedentary Behavior- Target for Change, Challenge to Assess	25	13:17	1056
66	http://www.youtube.com/watch?v=qondXFSjPPA	Sedentary Behaviour & Health: Is the Chair the Most Important Threat to Health in 21^st^ Century?	0	31:56	4
67	http://www.youtube.com/watch?v=rTRHkUuLON8	Sedentary Behaviour (Get off the couch)	14	2:34	233
68	http://www.youtube.com/watch?v=hWWHhvxYzXk	Sedentary Behaviour Researchers - A Guaranteed Standing Ovation	19	0:10	179
69	http://www.youtube.com/watch?v=gB33PRJttyU	Sedentary Behaviour: Not Even Once	14	2:17	252
70	http://www.youtube.com/watch?v=qondXFSjPPA	Sedentary Life—Barriers to Physical Activities	26	23:36	644
71	http://www.youtube.com/watch?v=8_xeukXJPbk	Sedentary Lifestyle	16	4:15	56
72	https://www.youtube.com/watch?v=5b064VsRiiY	Sedentary Lifestyle	1	1:45	81
73	https://www.youtube.com/watch?v=2lWnUnQTfxU	Sedentary lifestyle	12	4:17	17
74	https://www.youtube.com/watch?v=cWaVOq0AAAQ	Sedentary Lifestyle - Get Fit or Get Fat	36	2:29	1451
75	https://www.youtube.com/watch?v=F2PP-7vDJh0	Sedentary Lifestyle - It's Bad!!! This's How I solved My Problem of Chronic Sedentary Lifestyle - p1	27	10:39	244
76	https://www.youtube.com/watch?v=oVGi6jZ99ys	Sedentary Lifestyle and Obesity	20	2:20	3738
77	http://www.youtube.com/watch?v=-Y68CfN6oCk	Sedentary Lifestyle As Damaging As Smoking, Study Says	46	1:55	1651
78	https://www.youtube.com/watch?v=tupL_3uAZx0	Sedentary Lifestyle Doubles Disability Risk in Seniors, Study Finds	3	1:19	37
79	https://www.youtube.com/watch?v=MxURWgdEfuY	Sedentary lifestyle p2 -It's Bad!!! This's How I solved My Problem of Chronic Sedentary Lifestyle	28	4:52	127
80	https://www.youtube.com/watch?v=EUG_lUEJfUI	Sedentary Lifestyle Takes Toll on Health	3	2:01	76
81	https://www.youtube.com/watch?v=Q36IfFpzwqY	Sedentary to Active Lifestyle	79	4:55	883
82	https://www.youtube.com/watch?v=I9dC2ASKT8U	Should Sedentary Lifestyle Be Considered a Medical Condition	21	1:27	138
83	https://www.youtube.com/watch?v=5l8w3OWC4BM	Sitting Is the New Smoking??	3	2:17	2577
84	https://www.youtube.com/watch?v=Wl3U8DlGlyU	Sleekgeek Talks to Heath24	21	3:28	1643
85	https://www.youtube.com/watch?v=391MFsMJeyo	Steven Needs to Change his Sedentary Lifestyle.	52	3:57	608
86	https://www.youtube.com/watch?v=gotapi_c7H0	Stop Sitting Your Life Away	10	1:42	1294
87	https://www.youtube.com/watch?v=2oDi1n4Cdso	The American Sedentary Lifestyle	40	1:58	1061
88	http://www.youtube.com/watch?v=8h3HM9O2nQU	Thesis Defense	8	1:44:46	235
89	http://www.youtube.com/watch?v=-NAFN0tzjBE	To Good Health: Battling Chronic Diseases Episode 1	29	12:29	33
90	http://www.youtube.com/watch?v=ysxpX1I4VPY	Toddlers on the Move	0	1:43	22
91	https://www.youtube.com/watch?v=8r0tBE-TyxM	Top Health and Fitness	20	1:05	2796
92	http://www.youtube.com/watch?v=c2Sa1Gczhoc	Walk with Wellness	2	2:02	25
93	https://www.youtube.com/watch?v=FaQbrcJU150	Walking: Get the Word Out	15	1:17	535
94	http://www.youtube.com/watch?v=3lGrOyFpjTg	We are Leading Sedentary Lives, Says Health Minister	4	2:16	16
95	https://www.youtube.com/watch?v=_mNe_IF8ocg	What are Risk Factors Associated with Sedentary Lifestyle and Poor Nutritional Habits in Brazil?	35	1:18	263
96	https://www.youtube.com/watch?v=5i5-ox64reY	What can we do to Combat a Sedentary Lifestyle?	12	3:14	68
97	http://www.youtube.com/watch?v=SWnOGts8Oew	Your Chair Is Killing You - Ernesto Ramirez	39	5:15	842
98	http://www.youtube.com/watch?v=MK9QGGzeQe4	Zamzee Interview on KRON 4 News	19	4:45	301
99	https://www.youtube.com/watch?v=iXhEedeIRnM&list=PLbx2DInNN4q-ZXkOL23D5VQlNka4NfTwb	Physical Inactivity: The Biggest Public Health Problem of the 21^st^ Century	7	1:07:17	1542
100	https://www.youtube.com/watch?v=I-RkFDrFhF8&list=PLbx2DInNN4q-ZXkOL23D5VQlNka4NfTwb&index=2	Too Much TV Bad for Your Health?	35	1:49	5253
101	https://www.youtube.com/watch?v=dEqtySX0wSs&list=PLbx2DInNN4q8eXTUVJhFuTYTLxhmcK-mm	Inactivity Increases Heart Disease Risk | Heart Disease	9	1:22	707
102	https://www.youtube.com/watch?v=3LBbHbZ8jxk	Report Says: Physical Inactivity Kills 5 Million a Year	22	7:19	108
103	https://www.youtube.com/watch?v=zrp5sF0za40	Dr Rutledge Cause of Obesity: Excessive Calories/Lack of Exercise. Calories In and Calorie Out	51	5:51	720
104	https://www.youtube.com/watch?v=I9dC2ASKT8U	Should Sedentary Lifestyle Be Considered a Medical Condition	21	1:27	361
105	https://www.youtube.com/watch?v=kPNMuuY7jLU	The Relationship Between Physical Activity and Childhood Overweight and Obesity	42	1:55	84
106	https://www.youtube.com/watch?v=Ofg3UlxFVM0	Couch Potato Toddlers	38	1:09	635

**Table 2:** Location of origin for videos (N=106).

Country	Videos, n (%)
Australia	4 (3.7)
Barbados	1 (0.9)
Belgium	1 (0.9)
Canada	12 (11.3)
Colombia	1 (0.9)
Kenya	1 (0.9)
New Zealand	1 (0.9)
Russia	1 (0.9)
South Africa	1 (0.9)
Spain	2 (1.9)
Ukraine	1 (0.9)
United Kingdom	8 (7.6)
United States	44 (41.5)
Not available	28 (26.4)

### Video Features

The four search phrases (“sedentary behavior”, “sedentary behaviour”, “sedentary lifestyle”, and YouTube topic channel) and two search methods (sorted by relevance or views) generated 58.5% (62/106) duplicate results. Of the results that were unique to a single search phrase (41.5%, 44/106), “sedentary lifestyle” generated 65.9% (29/44), “sedentary behaviour” generated 20.5% (9/44), and “sedentary behavior” generated 13.6% (6/44).

Table 3 presents the means and interquartile ranges of video views, length, time since uploaded to the YouTube site, and quantity of comments posted by users. There was no relationship between number of views and video length (*r*=-.10, *P*>.05), or the search phrase (*r*=.08, *P*>.05). Similarly, no relationship was evident between search phrase and the source of the video (*r*=-.05, *P*>.05) or the activity type presented in the video (*r*=-.09, *P*>.05).

**Table 3:** Descriptive features of video results (N=106).

	Mean (SD)	25^th^ percentile	50^th^ percentile	75^th^ percentile
Views	1008.9 (2202)	44.5	239.0	917.5
Video length (minutes)	9:01 (16:31)	1:44	3:00	5:40
Time on YouTube (months)	19.2 (17.0)	6.0	15.0	27.5
Comments	1.7 (5.7)	0	1	1
Shares	1.2 (3.9)	0	0	1

### Activity Type

**Figure 3:**
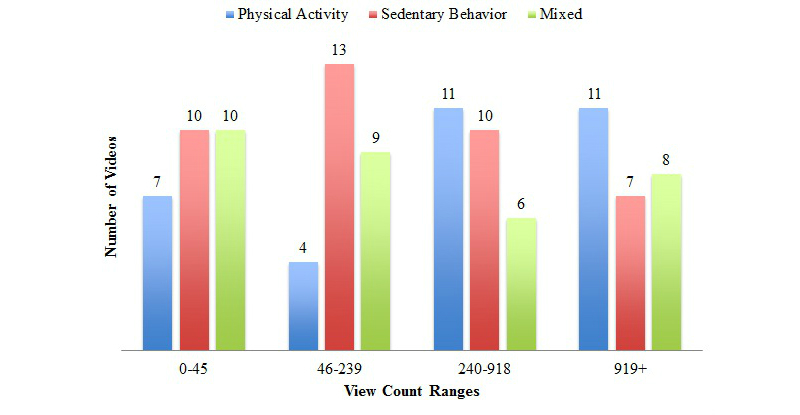
Activity type portrayed in videos by interquartile range of views.

Videos that portrayed content on sedentary behaviors alone represented 37.7% (40/106) of the results. Videos that portrayed a combination of sedentary and physical activity behaviors represented 31.1% (33/106) of the sample, and videos that portrayed physical activity behaviors alone similarly comprised 31.1% (33/106) of the sample. Videos with the highest view counts (ie >240 views) portrayed physical activity behavior content, not specifically sedentary behavior content (Figure 3).

### Source of the Content

**Figure 4:**
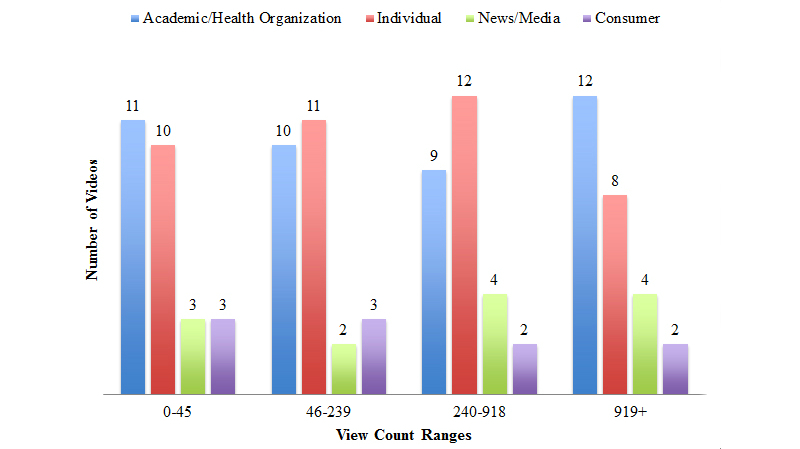
Source of videos presented by interquartile range of views.

Academic/Health Organizations and Individuals were the most common source of content, representing 39.6% (42/106) and 38.7% (41/106) of videos, respectively. News/Media comprised 12.3% (13/106) of the videos, and 9.4% (10/106) of videos were Consumer. The trend was similar across view count ranges (Figure 4).

### Purpose of the Content

**Figure 5:**
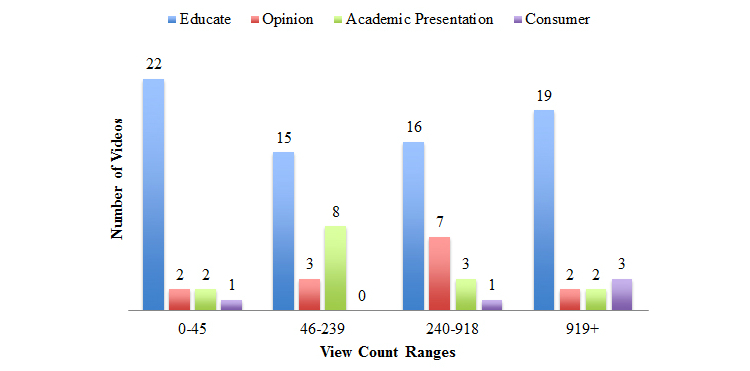
Purpose of video presented by interquartile range of views.

The predominant purpose of videos was coded as Educational (71/106, 67.0%). Academic presentations (16/106, 15.1%) and Opinions (14/106, 13.2%) contributed to a smaller portion of the available content. Minimal videos (5/106, 4.7%) were Consumer-based, aiming to sell products or services. Moreover, Educational videos were more dominant across all view count ranges (Figure 5).

##  Discussion

### Popularity of Content

YouTube is a popular social media site that facilitates the sharing of content, evidence-based or otherwise, with a large body of users. The purpose of this study was to explore descriptive features of sedentary behavior content on YouTube to enhance future knowledge translation efforts of evidence-informed sedentary behavior content. Results from 106 sedentary behavior videos demonstrate that content is being uploaded from around the world, which further underscores the value of this medium as a way to link the global community. Moreover, these results may underestimate the global reach of content via this social platform due to eligibility criteria from this study excluding videos not available in English.

Google AdWords [27] was used to generate a list of search phrases common to Internet users. The search phrase “sedentary lifestyle” generated more unique search results on YouTube than the other search phrases. However, no relationship was evident between search phrase and either views or source of the video. These results suggest that certain key terms are more prevalent on the site, yet this does not appear to impact viewership of sedentary behavior content on this social media platform.

To help design the future development of sedentary behavior content for YouTube, our investigation explored the relationship between length of videos and view counts. There was no relationship, suggesting that length of video does not impact the viewership. Therefore, knowledge translation efforts may need to consider aspects other than length of videos in order to increase viewership.

User-generated comments on videos represent the interactive and collaborative nature of YouTube content. The videos included this analysis had generated very few comments on the YouTube site (Table 3), which may indicate that sedentary behavior content currently on YouTube is not generating discussion and collaboration among users. Similarly, there were a remarkably low number of shares for sedentary behavior videos, indicating that users are not engaging in further social features of the YouTube site to enhance the reach of sedentary behavior content.

Moreover, view counts can be used to compare popularity of content on YouTube. Typically, popular videos on the site generate hundreds of thousands to millions of views. The median view count of sedentary behavior videos was 239 (Table 3), which suggests that the sedentary behavior content posted to the site is not popular among users. This presents a substantial opportunity for sedentary behavior researchers to improve the reach and impact of evidence through this social platform.

### Evidence-Based Content

In the past, the term “sedentary” was often used to refer to individuals who were not sufficiently physically active [4,29]. However, as noted above, available evidence suggests that sedentary behavior and physical activity should be viewed as separate and distinct constructs [4,30]. Thus, a growing number of researchers have suggested that the term “sedentary” should be used only to describe sedentary behaviors (eg, activities done while sitting), as opposed to the lack of physical activity [4].

Despite the widely used academic definition of sedentary behavior as activities characterized by sitting, approximately one third of this sample of videos displayed content on physical activity, not sedentary behaviors. Further, videos with higher view counts tended to portray information on physical activity, not sedentary behaviors (Figure 3). Finally, there was no relationship between search phrase and the type of activity behavior presented in the video (ie, sedentary behavior, physical activity, or mixed). These results further underscore the confusion between physical activity and sedentary behavior, which may impact knowledge users’ understanding of both the behaviors themselves and the associated health outcomes of these distinct behaviors.

### Limitations

The source and content of information is variable in this medium. There is currently a lack of standardized tools for assessing quality of content on social media sites like YouTube. Unlike systematic reviews of traditional evidence, a gap exists in the literature describing an evidence-based quality assessment tool for the purpose of reviewing social media content.

Moreover, previous research has highlighted concerns about the regulation of content available online. For example, one study that examined YouTube for evidence-based immunization content found that videos containing information that contradicted public health guidelines on the topic of interest were more likely to receive high view counts and user ratings and accounted for more than half of YouTube content on the topic [20]. Moreover, another study that examined YouTube for video content on eating disorders found that one third of videos glorified the unhealthy behaviors, and that these videos were more likely to have higher view counts than videos that discouraged the behavior [25]. These findings may further underscore the importance of the research community leveraging the popularity of YouTube as a knowledge translation vehicle to promote evidence-based information.

While YouTube is accessible around the world, and results from this study demonstrate content being uploaded from across the globe, many workplaces and educational institutions restrict user access to social media sites like YouTube. Restricted access may limit the impact of this medium for translation of evidence-based information to users. Research demonstrating the cultural value of YouTube [31,32] in conjunction with the potential of the social platform for sharing evidence-based content may be of value for informing the future development of policies governing access to social sites like YouTube.

### Conclusions

While physical activity is a well-established research domain, the focus on sedentary behavior research is much more recent. Therefore, many information seekers are not only unaware of the health consequences of prolonged sedentary behaviors, but also of the distinction between “too much sitting” and “not enough exercise”. Moreover, there is a shortage of evidence describing the implementation and translation of evidence-based sedentary behavior information into lay landscapes, which could further impede an individual’s understanding of this health risk.

Our study explored sedentary behavior content available on YouTube. Findings demonstrate that there is confusion between physical activity and sedentary behaviors, that content is being uploaded to the site from around the globe, that content is primarily from health organizations and individuals with the purpose of educating fellow users, but that low views and comments suggest that sedentary behavior content is relatively underutilized on YouTube. Future research may wish to leverage social platforms, such as YouTube, to facilitate implementation of evidence-based sedentary behavior content.
